# Hotspots for Disease-Causing Mutations in the Mitochondrial TIM23 Import Complex

**DOI:** 10.3390/genes15121534

**Published:** 2024-11-28

**Authors:** Sahil Jain, Eyal Paz, Abdussalam Azem

**Affiliations:** 1 School of Neurobiology, Biochemistry and Biophysics, George S. Wise Faculty of Life Sciences, Tel Aviv University, Tel Aviv 6997801, Israel; eyalpaz88@gmail.com (E.P.); azema@tauex.tau.ac.il (A.A.); 2 Bioinformatics Centre, Dr. D.Y. Patil Biotechnology and Bioinformatics Institute, Dr. D.Y. Patil Vidyapeeth, Pune 411033, India; 3 Sagol School of Neuroscience, Tel Aviv University, Tel Aviv 6997801, Israel

**Keywords:** mitochondrial protein import, rare genetic disorders, TIM23 complex, Timm50

## Abstract

The human mitochondrial proteome comprises approximately 1500 proteins, with only 13 being encoded by mitochondrial DNA. The remainder are encoded by the nuclear genome, translated by cytosolic ribosomes, and subsequently imported into and sorted within mitochondria. The process of mitochondria-destined protein import is mediated by several intricate protein complexes distributed among the four mitochondrial compartments. The focus of this mini-review is the translocase of the inner membrane 23 (TIM23) complex that assists in the import of ~60% of the mitochondrial proteome, which includes the majority of matrix proteins as well as some inner membrane and intermembrane space proteins. To date, numerous pathogenic mutations have been reported in the genes encoding various components of the TIM23 complex. These diseases exhibit mostly developmental and neurological defects at an early age. Interestingly, accumulating evidence supports the possibility that the gene for Tim50 represents a hotspot for disease-causing mutations among core TIM23 complex components, while genes for the mitochondrial Hsp70 protein (mortalin) and its J domain regulators represent hotspots for mutations affecting presequence translocase-associated motor (PAM) subunits. The potential mechanistic implications of the discovery of disease-causing mutations on the function of the TIM23 complex, in particular Tim50, are discussed.

## 1. Introduction

Mitochondria are endosymbiotic organelles comprising four sub-compartments, namely, the semipermeable mitochondrial outer membrane (OM), the aqueous intermembrane space (IMS), the impermeable mitochondrial inner membrane (IM), and the aqueous matrix [1,2,3,4]. The human mitochondrial matrix maintains 2–10 copies of a 16 kb circular genome containing 37 genes, which encode for 13 subunits of respiratory complexes, 22 mitochondrial tRNAs, and 2 rRNAs [1,5,6,7,8]. The human mitochondrial proteome comprises around 1500 proteins. Thus, the majority of mitochondrial proteins are encoded by the nuclear genome, cytoplasmically translated, and then imported into and sorted within the organelle [9,10,11,12]. Mitochondria are vital for the viability of essentially all eukaryotes, serving a vast number of functions, including ~80% of total cellular energy production [13], the metabolism of amino acids, lipids, and nucleotides, the biosynthesis of iron–sulfur (Fe–S) clusters and co-factors [9,14,15], the maintenance of calcium homeostasis [13], participation in various signaling processes, cellular differentiation, control of the cell cycle and cell growth [16], and quality control via mitophagy [17,18] and apoptosis [19]. The mitochondrial protein import machinery was suggested to play a role in cytosolic quality control by transporting aggregation-prone proteins into mitochondria, facilitating their degradation [20].

These diverse functions are performed by multiple protein machineries found in the different sub-compartments of mitochondria [14,21]. As the cellular demand for mitochondrial functions is known to vary along the life cycle of cells or in different tissues, the mitochondrial complexome is constantly dynamic, with the expression level of each protein in a given complex being regulated at the level of transcription, translation, import, folding, and degradation. Seven intricate protein complexes residing in different mitochondrial compartments play key roles in the maintenance and regulation of the mitochondrial proteome by mediating protein uptake from the cytosol [13,14,22]. The outer membrane contains three such complexes, namely, the translocase of the outer membrane (TOM) complex [23], the sorting and assembly (SAM) complex [24], and the mitochondrial import (MIM) complex [25]. The mitochondrial IMS assembly (MIA) complex is located in the IMS/IM. The translocase of the inner membrane 23 (TIM23) complex, the TIM22 complex [26], and the cytochrome oxidase assembly (OXA) complex all reside in the inner membrane [27].

The TOM complex, considered to be the general import pore for translocation across the outer membrane, comprises the Tom20 and Tom70/Tim71 primary receptors [28], the Tom22 central receptor [29], the Tom40 channel [30], and the smaller proteins Tom5, Tom6, and Tom7 [31,32]. The SAM complex facilitates the insertion of β-barrel-specific proteins and a majority of α-helical Tom proteins into the lipid bilayer [33]. The MIM complex, in corporation with the SAM complex, assists in the translocation of α-helical, signal-anchored, and tail-anchored proteins of the outer membrane [34]. The MIA complex facilitates the import of IMS proteins containing a mitochondrial IMS sorting signal presenting cysteine-based Cx_3_C or Cx_9_C motifs [30,35]. The TIM22 complex facilitates the insertion and assembly of multi-pass transmembrane proteins, particularly carrier proteins [26,36]. The OXA complex facilitates co-translational insertion of proteins into the inner membrane, particularly those involved in the respiratory chain [37,38,39]. The TIM23 complex is involved in the translocation of ~60% of the mitochondrial proteome, which comprises all of the matrix proteins, many IM proteins, and a few IMS proteins [40,41,42,43,44,45,46,47].

Due to the TIM23 complex’s central role in mitochondrial protein import and biogenesis, very few cases of TIM23 complex disease-causing mutations were discovered in the past. However, in recent years, accumulating studies suggest that mutations in components of the TIM23 complex, in particular the Tim50 protein, are the cause of several developmental and neurological impairments. This mini-review discusses the features of these disease-causing mutations.

## 2. The Yeast TIM23 and Human TIMM23 Complexes

**Figure 1:**
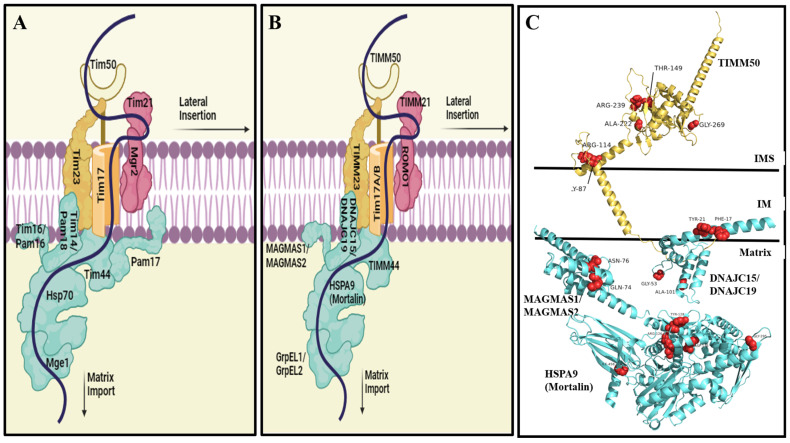
Panels (**A**,**B**) present schematic representations of the yeast and human TIM23 complexes, respectively. Core subunits are in yellow, subunits involved in lateral sorting of components are in pink, while PAM complex subunits are in sea-green. Notably, a human counterpart of yeast Pam17 is missing. A substrate protein of the complex is depicted in purple. In panel (**C**), all subunits of the TIM23 complex affected by documented mutations are presented. The structures for the indicated wild-type human subunits were predicted individually using AlphaFold. The predicted structures were visualized in PYMOL. The labelled residues are the wild-type residues which are affected by point mutations. The affected wild-type residues have been presented as spheres in red.

The TIM23 complex (also known as the presequence translocase) is the key player in the translocation of presequence-containing mitochondrial proteins [40,41,42,43,44,45,46,47]. Such proteins correspond to all of the matrix proteins, many IM proteins, and a few IMS proteins, representing in total ~60% of the mitochondrial proteome [40,41,42,43,44,45,46,47]. Current knowledge of the TIM23 complex is mainly based on studies conducted in yeast (Figure 1). The yeast TIM23 complex consists of the three membrane-associated core subunits: Tim23, Tim17, and Tim50 [1,48]. Association of these core TIM23 complex members with the Tim21 and Mgr2 subunits leads to the formation of the TIM23^SORT^ complex [41,49,50,51,52,53,54,55,56], which is implicated in the lateral insertion of a group of IM proteins [5] as well as the import and sorting of a few IMS proteins [57,58]. Alternatively, association of the TIM23^CORE^ complex with the PAM complex (comprising Tim44, Tim14/Pam18, Tim16/Pam16, mHsp70, and Mge1) leads to the formation of the TIM23^MOTOR^ complex [41,49,50,51,52,53,54,55,56], which is implicated in the import of matrix proteins as well as some IM proteins [59]. Notably, Pam17 is a yeast-specific subunit which fine-tunes the assembly of the PAM complex with the TIM23^CORE^ complex, and in doing so, helps mediate normal mitochondrial protein import [60]. The subunits of the different TIM23 complexes form highly dynamic entities, with the constant conformational changes that these proteins undergo being essential for normal complex function [41,45,61,62].

For many years, canonical models of import suggested that a Tim23 dimer forms a channel that serves as a path for protein import, while Tim17 acts as a regulatory element for channel opening and closure, maintaining membrane potential across the inner membrane. Recent cryo-electron microscopy and biochemical studies have, however, suggested that Tim17 monomers act as the main import path, with Tim23 likely serving as a regulatory element involved in structural maintenance [63,64,65]. It was further suggested that along with Tim17, Mgr2 plays a role in shielding precursor proteins from the lipid environment during translocation [64]. This role of Mgr2 supports the view that Tim23^SORT^ might be the first complex in the inner membrane to interact with the incoming precursor protein [30,66,67]. Indeed, a TOM–TIM23–precursor super-complex is not detected in the absence of Mgr2 [64]. Furthermore, Mgr2 was also suggested to be involved in the formation of the translocation path with Tim17 [63], although the involvement of Mgr2 in forming the translocation path is not crucial, as even in the absence of Mgr2 (a part of the TIM23^SORT^ complex), Tim17 can act as a channel for the Tim23^MOTOR^ pathway [63]. Indeed, precursor proteins reportedly progress to the Tim17 channel in the absence of Mgr2 [64]. Still, what triggers the formation/disruption of the Tim17-Mgr2 translocation path is currently unknown.

Unlike their yeast counterparts, human mitochondria contain multiple isoforms of presequence translocases, most probably to accommodate the multi-cellular nature of their hosts under normal or changing developmental stages [50]. In general, the components of the human TIM23 are similar to those of the yeast complex, albeit with the following differences:

1. In humans, due to the presence of isoforms of Tim17 and DnaJC (the homolog of yeast Tim14/Pam18), three TIMM23^MOTOR^ complex formations are presently known, namely, (i) translocase A consisting of Tim17a and DnaJC15, (ii) translocase B1 consisting of Tim17b_1_ and DnaJC19, and (iii) translocase B2 consisting of Tim17b_2_ and DnaJC19 [49,50]. Similar to the yeast complex, translocases B1 and B2 are expected to play major roles in protein import via the presequence pathway [50]. Interestingly, high expression of Tim17A mRNA has been reported in patients suffering from breast cancer [68,69]. Such elevated expression is closely linked to the aggressive growth of cancerous cells and adverse pathological and clinical outcomes [68,69]. Therefore, Tim17A is thought to be a prognostic biomarker for human breast cancer and a potential target for therapeutic developments.
2. The role played by Mgr2 in yeast mitochondrial protein import [64] is suggested to be carried out by ROMO1 (homolog of yeast Mgr2). However, this role has only been demonstrated for one protein, i.e., YME1L [70].
3. Interestingly, no homolog of Pam17 has yet been identified in humans. As discussed above, Pam17 plays a supportive role within the PAM complex. Although not essential, Pam17 is crucial for optimizing the activity of the import motor. Therefore, it would be worthwhile to identify those human subunits that fulfill the same function.
4. In yeast, Tim50 contains an extra, so-called presequence-binding domain (PBD) at its C-terminus (residues 395 to 476) thought to contribute to four major functions: (i) interaction with Tom22_IMS_ [22]; (ii) interaction with the presequence [71]; (iii) interaction with the conserved core domain of Tim50 (residues 164 to 361) [72]; and (iv) interaction with Tim21_IMS_ [73]. This suggests that Tim50 plays a highly dynamic and intricate role, especially the PBD, in IM and matrix protein import. However, in humans, Timm50 lacks the PBD. It is thus not surprising that human Timm50 was unable to complement its yeast homolog [74].

## 3. Genetic Variants in the TIM23 Complex

Given the central role played by mitochondrial import machineries in the maintenance of the mitochondrial proteome, it is expected that mutations that cause significant functional impairment in the import system will be lethal. Indeed, a small number of rare diseases have been identified in which mitochondrial import machineries are affected [75,76,77,78].

Notably, in the last decade, several mutations have been reported in genes encoding several subunits of the TIM23 complex (Table 1). Such rare mutations have been reported to cause various developmental and neurological conditions, including epileptic encephalopathy, infantile spasms, brain atrophy, developmental delay, and basal ganglia lesions. However, a direct mechanistic link between neurological symptoms and TIM23 complex-related mutations has yet to be established.

Table 1 summarizes all currently known genetic disease-causing mutations in genes encoding subunits of the TIM23 complex (Table 1). Supplementary Table S1 indicates the detection method of the mutations and provides an exhaustive list of all symptoms associated with each mutation.

Amongst the various subunits of the TIM23 complex, it is notable that no pathogenic mutations have been reported in genes encoding two core components (i.e., Timm23, and Timm17A/B). Remarkably, mutations have also not been reported in the genes of either of the lateral-sorting components (i.e., Timm21 and ROMO1; Table 1) or the motor components Tim44 and Mge1. This indicates either that mutations affecting these components are lethal or that they await discovery. Subunits implicated in genetic diseases include Timm50, Tim14 (DNAJC), Pam16 (Magmas), and mHsp70 (mortalin). The implications of the mutations in the genes encoding these proteins are discussed here.

### 3.1. Genetic Diseases Associated with Tim50

The highest number of mutations (eight mutations) has been reported to affect Tim50 (Timm50; Table 1) [74,75,79,80,81,82]. This is surprising, as in yeast, this subunit was shown to be essential for growth under all conditions and is thought to serve as the receptor subunit that receives precursor proteins as they emerge from the TOM complex [40,41,42,43,44,45,46,47]. Clinical reports indicate that the human Timm50 mutant characteristically exhibits several clinical features, including 3-methylglutaconic aciduria type 9 (i.e., high levels of 3-MGA in blood and urine) and failure to thrive in most cases [75,91]. Additionally, encephalopathy, the abnormality of visual evoked potentials, and an abnormal electroretinogram were reported in at least 60% of individuals with such mutations. Specifically, optic atrophy and elevated blood lactate levels were reported for R114W [75], T149M [74], and G269S [79]. A report by Shahrour et al. [74] indicated increased aggression in the patients (R114W and T149M), while Tort et al. [79] indicated strabismus, scoliosis, and piramidalism in R114Q patients. Interestingly, though normal Timm50 mRNA levels were detected in the case of two mutations (R114Q and G269S), lower Timm50 protein levels were still reported [79].

A structural examination of the mutated residues in the AlphaFold-derived complete Timm50 structure suggests that the R239W mutation is likely to be the most pathogenic, followed by R114W and T149M, while A222T, G269S, and R114Q are more likely to disrupt the structural integrity of the protein (Figure 1C; Supplementary Table S2). Overall, the corresponding mutations mostly lead to the structural destabilization or altered flexibility of Timm50, consequently suppressing its conformational changes that are important for TIM23 complex function.

Impaired protein import was reported in case of the G87A mutation [75]. An analysis of patient-derived fibroblasts demonstrated that Timm50 gene mutations lead to severe deficiency in the level of Timm50 protein [75,91,92]. Notably, this decrease was accompanied by a decrease in the level of two other core subunits, Tim23 and Tim17. However, unexpectedly, proteomics analysis in two recent studies indicated that the steady-state levels of most TIM23-dependent proteins were not affected by point mutations (Thr149Met and Arg113Cys), despite a drastic decrease in TIM23^CORE^ complex levels [91,92]. The proteins most affected were subunits of intricate complexes, such as the OXPHOS and ribosomal machineries [91,92]. Thus, both these studies indicate the surprising possibility that even a small fraction of functional TIM23 complex is able to maintain the steady-state levels of mitochondrial proteins. It is therefore conceivable that profound functional defects become evident only under import overload, such as during stress or under certain developmental conditions. The fact that mutations affecting Timm50 do not cause a global import defect explains why patients that carry these mutations survived.

### 3.2. Genetic Diseases Associated with mHsp70

mHsp70 (mortalin) is involved in numerous cellular processes including two major mitochondrial functions. mHsp70 serves as the heart of the PAM complex, mediating import across the inner membrane. It also mediates the disaggregation and refolding of newly imported and stress-denatured proteins with the aid of the non-membranal J-protein Tid1 [93,94,95]. Like other hsp70 proteins, mHsp70 is also expected to participate in mediating the degradation of misfolded proteins.

In the case of mortalin, five mutations have been reported to date (Table 1). Individuals carrying these mutations are characterized by anemia, iron overload, congenital malformations, and developmental issues. Characteristically, EVEN-plus syndrome is only observed in patients carrying mHsp70-specific mutations and not other TIM23-related mutations (Table 1). D136_I137insTer and I458_N459del reportedly led to a 50% and 80% decrease in mHsp70 mRNA and protein levels, respectively [89,90]. Arg126Trp and Tyr128Cys led to “bifid” distal femurs, arched eyebrows, and synophrys, while Tyr128Cys and Gly295_Val296insTer were observed to result in Atrioseptal Defect (ASD) and lateral vertebral clefts [78]. The symptoms listed above reflect the diverse impacts of mHsp70 gene mutations on both the hematological and developmental systems. However, reports on detailed clinical symptoms for other mHsp70 mutations are rather limited.

Structurally, mutations affecting residues 126, 128, 137, and 296 are expected to affect the stability and functionality of the mHsp70 ATPase domain (residues 1–360), thereby disrupting ATP binding and hydrolysis. As R126W and Y128C are the only known mHsp70 missense mutations, we analyzed their pathogenicity and effect on protein stability (Supplementary Table S2). The former is predicted to be more pathogenic, and the latter is predicted to destabilize the mHsp70 structure more. Specifically, R126W may lead to steric clashes due to the introduction of the bulky tryptophan side chain, while Y128C will affect protein stability through the loss of hydrogen bonds and hydrophobic interactions. Interestingly, Y128C was predicted to be the least pathogenic (Supplementary Table S2) among the known TIM23 complex missense mutations. Lastly, the p.Ile458_Asn459del mutation in the mHsp70 gene results in the deletion of two adjacent residues, isoleucine-458 and asparagine-459. As these residues are located in the C-terminal domain, which is involved in substrate binding, such deletion likely affects the chaperone activity of the protein. As mHsp70 plays a key role in both mitochondrial import and functions within mitochondria, it is currently unknown which lost function is responsible for disease manifestation.

### 3.3. Genetic Diseases Associated with Tim14 and Tim16

Tim14 (Pam18/DNAJC15/19), together with Tim16 (Pam16/Magmas), is believed to regulate the ATPase activity of mHsp70, thereby regulating its role in protein import. In the case of Tim14, five mutations have been reported in the encoding gene (Table 1), with 3-methylglutaconic aciduria type 5 and dilated cardiomyopathy being clinically diagnosed in all cases. Most of the mutations also cause decreases in steady-state Tim14 protein levels, growth failure, and hypotonia [84,85,87,96]. In particular, Tyr21Ter [84,85,86] and Ala101fs [87] have a detailed clinical description, with both leading to ataxia, short stature, optic atrophy, and gastrointestinal dysmotility. However, detailed clinical descriptions of other Tim14 mutations are limited. Overall, it may be concluded that Tim14 mutations lead to a range of neurological, developmental, metabolic, and systemic symptoms. Structurally, Conserved Domain Architecture Retrieval Tool (CDART) analysis indicates that a conserved DnaJ domain is located at the C-terminal of the protein (residues 66–116) [84]. Therefore, mutation affecting residue 101, found in the J domain, should affect protein functionality, possibly by disrupting its interaction with mortalin. Additionally, the Positioning of Proteins in Membranes (PPM) [97] webserver predicts that residues 4–23 comprise the Tim14 transmembrane domain (Figure 1C). Therefore, mutations affecting residues 17 and 21 would be expected to disrupt the membrane association of the Tim14 protein. Remarkably, Gly53Glu is the only missense mutation among all the Tim14 mutations and has the highest pathogenicity prediction (Supplementary Table S2) among all the known TIM23 complex mutations. This prediction, along with the multi-faceted symptoms, further supports the significance of the Tim14 protein and highlights the need for further experimental evaluation.

In addition to its import function, Pam16 (Magmas) was found to modulate granulocyte–macrophage colony-stimulating factor (GM-CSF) signaling [98] and may be important in reactive oxygen species (ROS) homeostasis [99]. In the case of Pam16, both known mutations (https://omim.org/entry/613320, accessed on 30 October 2024) in the encoding gene (Table 1) lead to characteristic developmental skeletal abnormalities, such as platyspondyly and reduced length of the long bones, as well as craniofacial dysmorphism [77,88]. These studies underscore the critical role of Pam16 in bone development, offering valuable insights into the genetic and clinical spectrum of skeletal dysplasias. Structurally, the Gln74Pro mutation introduces a proline, which would certainly disrupt helix_71–84_. Given its location and flexibility, this helix plausibly interacts with other import subunits and plays a role in the function of the protein. As such, its disruption is expected to lead to functional loss. Indeed, the mutation was predicted to be more pathogenic and less destabilizing (Supplementary Table S2). On the other hand, the Asn76Asp mutation is expected to disrupt hydrogen bonding and electrostatic interactions, thus affecting the stability of the protein. Indeed, the DUET webserver predicts this mutation to be highly destabilizing, with a −1.375 Kcal/mol change in energy (Supplementary Table S2).

Tim14 and Pam16 are thought to form a complex while fulfilling their roles. Yet, surprisingly, reports indicate that Tim14 gene mutations are more likely to reduce Tim14 protein levels, while Pam16 gene mutations are not reported to decrease steady-state Pam16 levels [77,84,85,86,88], and the disease-causing mutations result in different clinical developmental features.

## 4. Future Perspectives

The mitochondrial protein import system plays a key role in the biogenesis of mitochondrial proteins, and, thus, in mitochondrial functions. Initially, it was assumed that due to its vital functional role, any mutations affecting any of the central components of the different import systems would be lethal at the embryonic stage. Indeed, homozygous knockout Tim23 mice are not viable. However, in recent years, a growing body of evidence has shown that many rare genetic diseases result from mutations in genes encoding components of the import system. These mutations can serve as a highly valuable tool for research aimed at understanding the molecular mechanism of function of the mitochondrial import system. Notably, among the different TIM23 subunits, the Timm50 protein emerges as the hotspot for pathogenic mutations. Hence, research models featuring defective Timm50 can serve as potential screening models for therapeutic efforts aimed at mitochondrial import-associated disease. Such models can potentially be used to screen for small-molecule drugs aimed at symptomatic treatment, or even to test disease-modifying treatments such as gene therapy and mitochondrial augmentation.

## 5. Conclusions

Due to its vital role in the biogenesis of mitochondria, disease-associated mutations in the TIM23 complex are rare and are completely absent in several of its core subunits. Recent reports have indicated the presence of eight mutations in Timm50, some of which have been deeply investigated. The mechanistic outcome of these studies was that Timm50 is essential for the sorting of some of its putative substrates (lateral sorting). However, for some of the other components, only single cases were reported. Thus, our ability to extract the mechanistic implications of such mutations is limited.

## Figures and Tables

**Table 1:** Rare genetic diseases of the Timm23 complex.

Protein	Pathogenic/Likely Pathogenic Mutation *	Symptoms	ClinVar Accession No.	First Deposited in ClinVar	Reference
TIM23 core components					
Tim23	NA				
Tim17A	NA				
Tim17B	NA				
Tim50	NM_001001563.5(TIMM50):c.26C>A (p.Ser9Ter)	Mitochondrial encephalopathy, Reduced TIMM50 mRNA levels, OXPHOS malfunction, Failure to thrive, Lactic acidosis	RCV000677434.1, RCV001328000.2	24 August 2018	[75]
	NM_001001563.5(TIMM50):c.260G>C (p.Gly87Ala)	Mitochondrial encephalopathy, Reduced TIMM50 mRNA levels, OXPHOS malfunction, Failure to thrive, Lactic acidosis	RCV000677433.1, RCV001328001.1	24 August 2018	[75]
	NM_001001563.5(TIMM50):c.341G>A (p.Arg114Gln)	Encephalopathy, Decreased complex I, II, IV and V levels, Abnormality of visual evoked potentials, Strabismus, Scoliosis	RCV001812628.1, RCV003120700.4	19 January 2022	[79]
	NM_001001563.5(TIMM50):c.340C>T (p.Arg114Trp)	Epileptic encephalopathy, Decreased complex V activity, Elevated CSF lactate levels, Myoclonic jerks, Cachectic	RCV000509033.3, RCV001367110.6	9 October 2017	[74]
	NM_001001563.5(TIMM50):c.446C>T (p.Thr149Met)	Epileptic spasms, Hypsarrhythmia, Bilateral optic atrophy, Abnormal EEG, Developmental delay	RCV000509024.3	9 October 2017	[74,80]
	NM_001001563.5(TIMM50):c.664G>A (p.Ala222Thr)	3-methylglutaconic aciduria type 9	RCV000578358.5	8 February 2018	[81]
	NM_001001563.5(TIMM50):c.715C>T (p.Arg239Trp)	3-methylglutaconic aciduria type 9	RCV000578437.5, RCV002529040.2	8 February 2018	[82]
	NM_001001563.5(TIMM50):c.805G>A (p.Gly269Ser)	Encephalopathy, Failure to thrive, Spastic tetraparesia with dystonia, Piramidalism, Elevated CSF lactate levels	RCV000190713.5, RCV001812182.1	14 September 2015	[79]
TIM23 lateral-sorting components					
Tim21	NA				
Mgr2	NA				
TIM23 motor components/PAM complex					
Tim44	NA				
Tim14 (Isoform 1)	NM_145261.4(DNAJC19):c.51del (p.Phe17fs)	Dilated cardiomyopathy with ataxia, Lipidosis, 3-methylglutaconic aciduria type 5	RCV001231277.9	16 July 2020	[83,84,85]
	NM_145261.4(DNAJC19):c.63del (p.Arg20_Tyr21insTer)	Dilated cardiomyopathy with ataxia, 3-methylglutaconic aciduria type 5	RCV001780991.5	29 November 2021	[84,85]
	NM_145261.4(DNAJC19):c.63C>G (p.Tyr21Ter)	Dilated cardiomyopathy with ataxia, Failure to thrive, Optic atrophy, 3-methylglutaconic aciduria type 5, 3-methylglutaconic aciduria type 3	RCV001206673.7, RCV001824933.1	16 July 2020	[84,85]
	NM_145261.4(DNAJC19):c.62dup (p.Tyr21Ter)	Dilated cardiomyopathy with ataxia, 3-methylglutaconic aciduria type 5	RCV001729987.3	16 October 2021	[84,85,86]
	NM_145261.4(DNAJC19):c.158G>A (p.Gly53Glu)	Dilated cardiomyopathy with ataxia, 3-methylglutaconic aciduria type 5	RCV001283818.1	26 January 2021	[76]
	NM_145261.4(DNAJC19):c.300del (p.Ala101fs)	Dilated cardiomyopathy with ataxia, Noncompaction cardiomyopathy, 3-methylglutaconic aciduria type 5	RCV000106304.5	24 March 2014	[87]
Tim14 (Isoform 2)	NA				
Pam16	NM_016069.11(PAM16):c.221A>C (p.Gln74Pro)	Autosomal recessive spondylometaphyseal dysplasia, Megarbane type, Macrocephaly, Developmental delay, Hypotonia, Narrow spinal cord	RCV000788051.3	22 July 2019	[77]
	NM_016069.11(PAM16):c.226A>G (p.Asn76Asp)	Autosomal recessive spondylometaphyseal dysplasia, Megarbane type, Developmental delay, Prominent abdomen, Square iliac bones, Respiratory insufficiency	RCV000167551.4	29 March 2015	[88]
mHsp70	NM_004134.7(HSPA9):c.376C>T (p.Arg126Trp)	EVEN-plus syndrome (EVPLS) [Epiphyseal and vertebral dysplasia, microtia, and flat nose, plus associated malformations]	RCV000210028.3	14 March 2016	[78]
	NM_004134.7(HSPA9):c.383A>G (p.Tyr128Cys)	EVEN-plus syndrome (EVPLS) [Epiphyseal and vertebral dysplasia, microtia, and flat nose, plus associated malformations], Premature termination predicted to abolish half the protein	RCV000209966.4	14 March 2016	[78]
	NM_004134.7(HSPA9):c.409_410del (p.Asp136_Ile137insTer)	Autosomal dominant sideroblastic anemia, 50% of HSPA9 mRNA and 80% of HSPA9 protein	RCV000209839.4	12 March 2016	[89,90]
	NM_004134.7(HSPA9):c.882_883del (p.Gly295_Val296insTer)	EVEN-plus syndrome (EVPLS) [Epiphyseal and vertebral dysplasia, microtia, and flat nose, plus associated malformations], Predicted to result in premature protein termination, Developmental delay	RCV000209995.6, RCV001781629.4, RCV003387515.2	12 March 2016	[78]
	NM_004134.7(HSPA9):c.1373_1378del (p.Ile458_Asn459del)	Autosomal dominant sideroblastic anemia, 50% of HSPA9 mRNA and 80% of HSPA9 protein	RCV000209862.4	12 March 2016	[89]
Mge 1 (Isoform 1)	NA				
Mge 1 (Isoform 2)_	NA				

* Only “Reviewed” UniProt entries (found in Swiss-Prot) were considered for each subunit. Also, only mutations that are reported to occur inside the gene and are reported as pathogenic were considered. Mutations of uncertain clinical significance were ignored.

## Supplementary Materials

The following supporting information can be downloaded at: https://www.mdpi.com/article/10.3390/genes15121534/s1, Table S1. Rare genetic diseases of the Timm23 complex; Table S2. An estimation of the pathogenicity (AlphaMissense) and stability changes (DUET) of the various missense mutations reported in the TIM23 complex subunits.
