# Supplementary material for: Hotspots for Disease-Causing Mutations in the Mitochondrial TIM23 Import Complex
Source: Genes (Basel). 2024 Nov 28;15(12):1534. doi: 10.3390/genes15121534 (PMC11675802; doi:10.3390/genes15121534)
Supplement: Supplementary file 1 [file genes-15-01534-s001.zip › Supplementary Table S1.pdf]

Supplementary Table S1. Rare genetic diseases of the Timm23 complex.

Protein	Pathogenic/Likely Pathogenic mutation	Symptoms	Detection Technique
TIM23 core components			
**Tim23**	NA		
**Tim17A**	NA		
**Tim17B**	NA		
**Tim50**	NM_001001563.5(TIMM50):c.26C>A (p.Ser9Ter)	Mitochondrial encephalopathy, Reduced TIMM50 mRNA levels, Lower mitochondrial membrane potential, OXPHOS malfunction, High ROS production, Neonatal hypotonia, Failure to thrive, Infantile spasms, Hypsarrhythmia, Lactic acidosis, Hyper-beta-alaninemia, Abnormality of visual evoked potentials, Abnormal electroretinogram, Brain atrophy, Respiratory arrest, 3-methylglutaconic aciduria type 9	Whole Exome Sequencing (WES)
	NM_001001563.5(TIMM50):c.260G>C (p.Gly87Ala)	Mitochondrial encephalopathy, Reduced TIMM50 mRNA levels, Lower mitochondrial membrane potential, OXPHOS malfunction, High ROS production, Neonatal hypotonia, Failure to thrive, Infantile spasms, Hypsarrhythmia, Lactic acidosis, Hyper-beta-alaninemia, Abnormality of visual evoked potentials, Abnormal electroretinogram, Brain atrophy, Respiratory arrest, 3-methylglutaconic aciduria type 9	WES
	NM_001001563.5(TIMM50):c.341G>A (p.Arg114Gln)	Encephalopathy, Lower TIMM50 protein levels, Decreased complex I, II, IV and V levels, Failure to thrive, Abnormality of visual evoked potentials, Abnormal electroretinogram, 3-methylglutaconic aciduria type 9, Abnormal mito cristae structure, Neurological regression, Dilated cardiomyopathy, Strabismus, Scoliosis, Spastic tetraparesia with dystonia, Piramidalism, Osteoarticular problems, Wheelchair-dependent, Elevated blood lactate levels, Elevated CSF lactate levels, Aggregation of lipidic material in muscle fibers	WES
	NM_001001563.5(TIMM50):c.340C>T (p.Arg114Trp)	Epileptic encephalopathy, Decreased complex V activity, Failure to thrive, 3-methylglutaconic aciduria type 9, Elevated CSF lactate levels, Myoclonic jerks, Cachectic, Abnormal EEG, Delayed psychomotor development, Hyperactive, Aggressive when frightened, Generalized tonic movements of the upper and lower limbs, Uprolling of the eyes, Severely reduced muscle mass, Increased muscle tone, Supratentorial atrophy	WES
	NM_001001563.5(TIMM50):c.446C>T (p.Thr149Met)	Epileptic spasms, Severe hypotonia, Failure to thrive, Infantile spasms, Hypsarrhythmia, 3-methylglutaconic aciduria type 9, Retarded, Bilateral optic atrophy, Abnormal EEG, Elevated plasma lactate levels, Aggressive behavior, Involuntary abnormal movements, Poor communication, Bilateral acetabular dysplasia, Developmental delay	WES
	NM_001001563.5(TIMM50):c.664G>A (p.Ala222Thr)	3-methylglutaconic aciduria type 9	Unknown
	NM_001001563.5(TIMM50):c.715C>T (p.Arg239Trp)	3-methylglutaconic aciduria type 9	Unknown

	NM_001001563.5(TIMM50):c.805G>A (p.Gly269Ser)	Encephalopathy, Lower TIMM50 protein levels, Decreased complex I, II, IV and V levels, Failure to thrive, Abnormality of visual evoked potentials, Abnormal electroretinogram, 3-methylglutaconic aciduria type 9, Abnormal mito cristae structure, Neurological regression, Spastic tetraparesia with dystonia, Piramidalism, Elevated blood lactate levels, Elevated CSF lactate levels, Scoliosis, Osteoarticular problems, Wheelchair-dependent, Aggregation of lipidic material in muscle fibers	WES
TIM23 lateral sorting components			
**Tim21**	NA		
**Mgr2**	NA		
TIM23 motor components / PAM complex			
**Tim44**	NA		
**Tim14 (Isoform 1)**	NM_145261.4(DNAJC19):c.51del (p.Phe17fs)	Lower or no DNAJC19 levels, Lipidosis, Dilated cardiomyopathy with ataxia, Lipidosis, 3-methylglutaconic aciduria type 5	Next Generation Sequencing (NGS)
	NM_145261.4(DNAJC19):c.63del (p.Arg20_Tyr21insTer)	Lower or no DNAJC19 levels, Dilated cardiomyopathy with ataxia, 3-methylglutaconic aciduria type 5	Sanger sequencing
	NM_145261.4(DNAJC19):c.63C>G (p.Tyr21Ter)	Lower or no DNAJC19 levels, Abnormal retinal morphology, Dilated cardiomyopathy with ataxia, Failure to thrive, Optic atrophy, Abnormal EEG, Movement disorder, Abnormal muscle physiology (limbs, pelvis), Developmental dysplasia of the hip, Sensorineural deafness, Basal ganglia lesions, Short stature, Abnormal hair morphology, Abnormal oral cavity morphology, Oral-pharyngeal dysphagia, Myopia, Hearing impairment, Tinnitus, Vertigo, Depression, Hyperpigmentation of the skin, Hyperhidrosis, Cardiac arrhythmia, Abnormal esophagus morphology, Abnormal stomach morphology, Gastrointestinal dysmotility, Abnormality of the bladder, Abnormal inflammatory response, bruising susceptibility, tooth malposition, 3-methylglutaconic aciduria type 5, 3-methylglutaconic aciduria type 3	Homozygosity mapping approach
	NM_145261.4(DNAJC19):c.62dup (p.Tyr21Ter)	Lower or no DNAJC19 levels, Dilated cardiomyopathy with ataxia, 3-methylglutaconic aciduria type 5	Unknown
	NM_145261.4(DNAJC19):c.158G>A (p.Gly53Glu)	Dilated cardiomyopathy with ataxia, 3-methylglutaconic aciduria type 5	Unknown
	NM_145261.4(DNAJC19):c.300del (p.Ala101fs)	No detectable DNAJC19 levels, Dilated cardiomyopathy with ataxia, Noncompaction cardiomyopathy, 3-methylglutaconic aciduria type 5, neonatal hypoglycemia, slight anisocytosis, elevated plasma alanine aminotransferase, developmental delay, mild ataxia, respiratory chain enzyme deficiency in skeletal muscle, enlarged left ventricle, perfused intertrabecular recesses	Sequencing
**Tim14 (Isoform 2)**	NA		

**Pam16**	NM_016069.11(PAM16):c.221A>C (p.Gln74Pro)	Autosomal recessive spondylometaphyseal dysplasia, Megarbane type, macrocephaly, depressed nasal bridge, prominent abdomen, developmental delay, hypotonia, narrow spinal cord, atlantoaxial instability	WES
	NM_016069.11(PAM16):c.226A>G (p.Asn76Asp)	Autosomal recessive spondylometaphyseal dysplasia, Megarbane type, developmental delay, growth retardation, narrow thorax, short ribs, short limbs, prominent abdomen, square iliac bones, horizontal acetabulae, respiratory insufficiency	WES
**mHsp70**	NM_004134.7(HSPA9):c.376C>T (p.Arg126Trp)	Even-plus syndrome (EVPLS) [Epiphyseal and vertebral dysplasia, microtia, and flat nose, plus associated malformations], short long bones, severe bilateral microtia, arched eyebrows, mild synophris, aplasia cutis, dysplasia of the femoral heads, “bifid” distal femurs, dysplastic knee epiphyses, oligohydramnios, rectal atresia without fistula, developmental delay, persistent foramen ovale, aneurysmatic septum, brachycephaly	WES
	NM_004134.7(HSPA9):c.383A>G (p.Tyr128Cys)	Even-plus syndrome (EVPLS) [Epiphyseal and vertebral dysplasia, microtia, and flat nose, plus associated malformations], hypoplastic nasal bones, arched eyebrows, synophrys, small and poorly formed ears, anal atresia, atrioseptal defect (ASD), lateral vertebral clefts, “bifid” distal femurs, Premature termination predicted to abolish half the protein	WES
	NM_004134.7(HSPA9):c.409_410del (p.Asp136_Ile137insTer)	Autosomal dominant sideroblastic anemia, 50% of HSPA9 mRNA and 80% of HSPA9 protein	Sanger Sequencing
	NM_004134.7(HSPA9):c.882_883del (p.Gly295_Val296insTer)	Even-plus syndrome (EVPLS) [Epiphyseal and vertebral dysplasia, microtia, and flat nose, plus associated malformations], hypoplastic nasal bones, arched eyebrows, synophrys, small and poorly formed ears, anal atresia, atrioseptal defect (ASD), lateral vertebral clefts, “bifid” distal femurs, Premature termination predicted to abolish half the protein	WES
	NM_004134.7(HSPA9):c.1373_1378del (p.Ile458_Asn459del)	Autosomal dominant sideroblastic anemia, 50% of HSPA9 mRNA and 80% of HSPA9 protein	Sanger Sequencing
**Mge 1 (Isoform 1)**	NA		
**Mge 1 (Isoform 2)**	NA		

*Only “Reviewed” UniProt entries (found in Swiss-Prot) were considered for each subunit. Also, only mutations that are reported to occur inside the gene and as pathogenic were considered. Mutations of uncertain clinical significance were ignored.
