# Supplementary material for: Hotspots for Disease-Causing Mutations in the Mitochondrial TIM23 Import Complex
Source: Genes (Basel). 2024 Nov 28;15(12):1534. doi: 10.3390/genes15121534 (PMC11675802; doi:10.3390/genes15121534)
Supplement: Supplementary file 1 [file genes-15-01534-s001.zip › Supplementary Table S2.pdf]

**Supplementary Table S2.** An estimation of the pathogenicity (AlphaMissense) and stability changes (DUET) of the various missense mutations reported in the TIM23 complex subunits.

| Protein | Pathogenic/Likely Pathogenic Missense Mutations | AlphaMissense Score | DUET Score (kcal/mol)* |
|---|---|---|---|
| *TIM23 core components* | | | |
| **Tim23** | NA | | |
| **Tim17A** | NA | | |
| **Tim17B** | NA | | |
| **Tim50** | NM_001001563.5(TIMM50):c.260G>C (p.Gly87Ala) | 0.81 | -0.233 |
| | NM_001001563.5(TIMM50):c.341G>A (p.Arg114Gln) | 0.888 | -1.658 |
| | NM_001001563.5(TIMM50):c.340C>T (p.Arg114Trp) | 0.963 | -0.94 |
| | NM_001001563.5(TIMM50):c.446C>T (p.Thr149Met) | 0.96 | -0.769 |
| | NM_001001563.5(TIMM50):c.664G>A (p.Ala222Thr) | 0.808 | -1.847 |
| | NM_001001563.5(TIMM50):c.715C>T (p.Arg239Trp) | 0.986 | -0.219 |
| | NM_001001563.5(TIMM50):c.805G>A (p.Gly269Ser) | 0.926 | -1.76 |
| *TIM23 lateral sorting components* | | | |
| **Tim21** | NA | | |
| **Mgr2** | NA | | |
| *TIM23 motor components / PAM complex* | | | |
| **Tim44** | NA | | |
| **Tim14 (Isoform 1)** | NM_145261.4(DNAJC19):c.158G>A (p.Gly53Glu) | 0.997 | -0.508 |
| **Tim14 (Isoform 2)** | NA | | |
| **Pam16** | NM_016069.11(PAM16):c.221A>C (p.Gln74Pro) | 0.73 | -0.114 |
| | NM_016069.11(PAM16):c.226A>G (p.Asn76Asp) | 0.447 | -1.375 |
| **mHsp70** | NM_004134.7(HSPA9):c.376C>T (p.Arg126Trp) | 0.93 | -0.374 |
| | NM_004134.7(HSPA9):c.383A>G (p.Tyr128Cys) | 0.248 | -1.218 |
| **Mge 1 (Isoform 1)** | NA | | |
| **Mge 1 (Isoform 2)** | NA | | |

*The AlphaFold structures, predicted for the structural representation of mutated residues, were used as input for this server.
